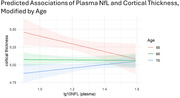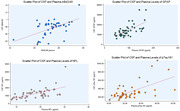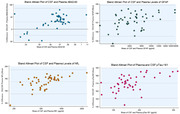# Association of Plasma Biomarkers of Amyloid, Neurodegeneration and Neuroinflammation, with Cognitive Performance, Cortical Thickness and Cerebrospinal Fluid Biomarkers

**DOI:** 10.1002/alz.087201

**Published:** 2025-01-09

**Authors:** Ming Ann Sim, Ella Rowsthorn, Will O'Brien, Lachlan Cribb, Katherine H. Franks, Stuart McDonald, Stephanie Yiallourou, Marina G. Cavuoto, Ian H Harding, Trevor T.‐J. Chong, Lucy Vivash, Terence J O’Brien, Meng Law, Matthew P. Pase

**Affiliations:** ^1^ Turner Institute for Brain and Mental Health & School of Psychological Sciences, Monash University, Clayton, VIC Australia; ^2^ National University of Singapore, Singapore, singapore Singapore; ^3^ Central Clinical School, Monash University, Melbourne, VIC Australia; ^4^ Monash University, Melbourne, VIC Australia; ^5^ Turner Institute for Brain and Mental Health, Monash University, Melbourne, VIC Australia; ^6^ Alfred Hospital, Melbourne, VIC Australia; ^7^ Monash University, Clayton, VIC Australia; ^8^ Alfred Health, Victoria, Melbourne Singapore

## Abstract

**Background:**

Plasma and cerebrospinal (CSF) biomarkers are promising candidates for detecting neuropathology. While CSF biomarkers directly reflect pathophysiological processes within the central nervous system, their requirement for a lumbar puncture is a barrier to their widespread scalability in practice. Therefore, we examined cross‐sectional associations of plasma biomarkers of amyloid (Aβ42/Aβ40 and pTau‐181), neurodegeneration (Neurofilament Light, NfL), and neuroinflammation (Glial Fibrillary Acidic Protein, GFAP) with brain volume, cognition, and their corresponding CSF levels.

**Method:**

This was a cross‐sectional dementia‐free community‐based cohort of participants aged ≥55 years. All participants underwent neuropsychological testing comprising mini‐mental state examinations (MMSE), clinical dementia rating sum‐of‐boxes (CDR‐SB), and domain‐specific tests (episodic memory, visual memory, language, and executive function, expressed as Z‐scores). SIMOA‐measured® paired CSF and plasma p‐Tau181, GFAP, Aβ42/Aβ40 ratio, and NfL were obtained, as were brain MRI scans. Multivariable linear regression adjusting for age, sex, and education was employed to evaluate associations of log_10_‐transformed plasma biomarker levels with cognitive performance and cortical thickness (adjusted for total intracranial volume). Pearson’s correlations and Bland‐Altman plots were employed to evaluate correlations and agreement between paired levels of CSF and plasma biomarkers.

**Result:**

Of 147 dementia‐free participants (mean age 66.7±7.7 years, 93% Caucasian), 34% were male.

Lower plasma Aβ42/40 was significantly associated with poorer CDR‐SB (β‐6.62, (95%C.I. ‐11.97, ‐1.27), p=0.016). Higher plasma GFAP levels were associated with lower MMSE (β‐1.70, (95%C.I.‐3.22, ‐0.18) p=0.029)). Higher plasma GFAP and NfL were significantly associated with poorer visual and episodic memory Z‐scores (all p<0.05). Lower plasma NfL levels were associated with greater cortical thickness (β‐3.43, 95%C.I. ‐6.18, ‐0.67, p=0.015), with significant interactions observed with age (p‐interaction=0.014, Figure 1).

Of 47 participants with CSF biomarkers, paired levels of plasma and CSF pTau‐181 (R:0.61, p<0.0001), Aβ42/40 (R:0.53, p=0.0001), GFAP (R:0.66, p<0.0001) and NfL (R:0.56, p<0.0001) were significantly correlated (Figure 2). Using Bland‐Altman analysis, greater dispersion was observed between paired CSF and plasma p‐Tau181 and Aβ42/40 levels (Figure 3).

**Conclusion:**

Plasma biomarkers of neurodegenerative, neuroinflammation and amyloid pathology related closely with cognitive performance, neuroimaging indices, and their paired CSF levels. These may serve as promising candidates for detecting early neuropathology even in a dementia‐free community‐based population.